# *PMEL* governs autosomal dominant inheritance of white-tail independent of yellow body plumage in chickens (*Gallus gallus domesticus*)

**DOI:** 10.1016/j.psj.2025.106127

**Published:** 2025-11-16

**Authors:** Xunhe Huang, Luheng Zhang, Zhipeng Zhong, Zhuoxian Weng, Yongjie Xu, Jintian Wen, Cheng Ma, Bingwang Du

**Affiliations:** a Guangdong Provincial Key Laboratory of Conservation and Precision Utilization of Characteristic Agricultural Resources in Mountainous Areas, Guangdong Innovation Centre for Science and Technology of Wuhua Yellow Chicken, School of Life Sciences, Jiaying University, 514015 Meizhou, China; b Department of Medical Biochemistry and Microbiology, Uppsala University, Uppsala, Sweden

**Keywords:** *PMEL*, Regional pigmentation, Tail feather, Autosomal dominance, Melanogenesis

## Abstract

Region-specific feather pigmentation is widespread in birds, yet the genetic mechanisms enabling independent control of body and tail plumage remain poorly understood. The white-tailed (WT) phenotype in Wuhua yellow chickens, which exhibit yellow body plumage but pure white tail feathers, provides an ideal model for dissecting such modular genetic regulation. Genetic analysis of F_1_ and F_2_ populations (*n* = 137) derived from a cross with Huaixiang (black-tailed, BT) chickens revealed an autosomal dominant inheritance pattern for the WT trait, with a 3:1 segregation ratio (105 WT: 32 BT; χ^2^ = 0.20, *P* = 0.66) in the F_2_ generation—independent of the yellow body plumage locus. Integrating genome-wide association study of 47 F_2_ roosters with population resequencing (*n* = 50), we identified a highly significant locus on chromosome 33 (*P* = 1.35E−8) harboring *PMEL* as the sole credible candidate. Tail follicle transcriptome profiling (4 WT vs. 4 BT) uncovered 1,712 differentially expressed genes, with coordinated downregulation of the entire melanogenesis pathway (normalized enrichment score = −1.914, FDR = 0.003). *PMEL* ranked among the most strongly suppressed genes (log_2_FC = −2.18, FDR = 5.58E−06). Furthermore, genome-wide alternative splicing analysis identified 2,764 significant events among 30,473 detected, indicating widespread post-transcriptional regulation. Consistent with this global pattern, the *PMEL* locus exhibited markedly reduced splicing complexity in WT chickens, characterized by a systematic shift toward simpler isoforms. Thus, four orthogonal lines of evidence—genomic association, transcriptional repression, pathway-level melanogenesis shutdown, and splicing simplification—converge on *PMEL* as the causal gene underlying this autosomal dominant, tail-specific depigmentation. Our findings establish *PMEL* as the primary regulator underlying this autosomal dominant trait, paving the way for understanding modular avian coloration and enabling its application in precision breeding programs.

## Introduction

Avian plumage pigmentation has long served as a model for understanding the genetic regulation of phenotypic traits, offering insights into developmental biology, evolutionary genetics, and animal breeding (Cooke et al., 2017; Price-Waldman and Stoddard, 2021). Feather coloration in birds is primarily determined by two melanin pigments: eumelanin (black/brown) and pheomelanin (red/yellow) (Elkin et al., 2023). Their genetic control involves a complex network, including the melanocortin system (*MC1R*/*ASIP*), melanogenic enzymes (*TYR, TYRP1, DCT*), and melanosome structural proteins (*PMEL*) (Andersson et al., 2020). The *MC1R*-*MITF* axis functions as the central regulatory hub, where *MC1R* activation triggers cAMP-dependent *MITF* expression, subsequently modulating pigment enzyme activity and melanosome biogenesis (Kawakami and Fisher, 2017). This pathway is evolutionarily conserved across vertebrates yet exhibits remarkable spatiotemporal flexibility, enabling intricate color patterns (Kratochwil and Mallarino, 2023).

Regional specialization of pigmentation represents a fundamental yet poorly understood aspect of color patterning (Popadic and Tsitlakidou, 2021; Kratochwil and Mallarino, 2023). In chickens, compartmentalized body-versus-tail coloration has emerged as a tractable paradigm. For example, ectopic agouti signaling protein (*ASIP*) gene overexpression underlies the yellow-body/black-tail phenotype in Huiyang bearded chickens (Zheng et al., 2024). Beyond transcriptional control, post-transcriptional mechanisms are increasingly implicated. This is exemplified in llamas, where alternative splicing (AS) of the *ASIP* gene generates multiple transcripts with alternative 5′UTRs, regulating spatial expression, and thereby directly influencing regional pigmentation patterns (Anello et al., 2022). Similarly, in WugangTong geese, a 14-bp insertion in Endothelin2 (*EDNRB2*) induces exon skipping, a specific form of AS, which leads to melanocyte loss in breast patches (Yang et al., 2024). Spatial pre-patterning can also be established embryonically, as exemplified by feline *DKK4*–*Taqpep*-mediated reaction–diffusion dynamics that template tabby stripes (Kaelin et al., 2021). Finally, PMEL itself contributes to regional fidelity through stage- and tissue-specific isoforms that compartmentalize melanin deposition (D’Alba and Shawkey, 2019). Overall, these examples illustrate how global pigment pathways can be spatially modulated at multiple regulatory levels, ranging from pre-patterning and transcriptional control to post-transcriptional splicing and melanosome biogenesis, to generate localized color differences.

The genetic basis of white and black plumage has been extensively investigated in avian species. White plumage typically results from mutations disrupting melanosome biogenesis (*PMEL*), melanocyte migration (*KIT*), or melanin synthesis (*SLC45A2*), whereas black plumage is often associated with enhanced eumelanin production via *MC1R* activation or *ASIP* suppression (Wen et al., 2021; Zhang et al., 2023; Horecka et al., 2024; Zheng et al., 2024; Wang et al., 2025b). Strikingly, tail-feather color can segregate independently of body plumage, implying modular genetic control (Nie et al., 2021; Zheng et al., 2024). A salient example is the Dominant White (I) allele at *PMEL* in White Leghorns, which selectively abolishes eumelanin in tail feathers while sparing yellow body plumage (Zheng et al., 2024). However, whether this tail-specific effect operates universally across breeds—and the extent to which post-transcriptional mechanisms enforce spatial restriction—remains unresolved.

Despite these advances, critical gaps remain in understanding region-specific pigmentation. Zheng et al. (2024) identified *ASIP*’s role in black-tailed (**BT**) Huiyang bearded chickens through follicular RNA analysis. Still, their study exclusively examined BT F_2_ individuals, leaving the molecular basis of white-tailed (**WT**) variants uncharacterized. This underscores the need to validate *PMEL*’s role in WT phenotypes across breeds, such as Wuhua yellow chickens, which exhibit yellow body plumage with a white-tail. Preliminary selective sweep analyses implicate both *PMEL* and *TYRP1* in Wuhua yellow chickens (Weng et al., 2020), suggesting potential breed-specific genetic pathways for this trait. Importantly, the mechanisms by which *PMEL* to selectively inhibits melanogenesis in tail feathers while preserving body pigmentation remain poorly understood, particularly at the post-transcriptional level.

To address these questions, we conducted a systematic genetic analysis of Wuhua yellow chicken (yellow body/white tail) and Huaixiang chickens (yellow body/black tail). We hypothesized that body-versus-tail pigmentation is governed by autonomous genetic loci acting through coordinated transcriptional and post-transcriptional reprogramming. We specifically tested whether *PMEL* underlies this dominant, tail-restricted depigmentation and how splicing modulation enforces regional specificity. Our objectives were threefold: (1) establish the inheritance pattern in a novel mapping population; (2) pinpoint the causal locus via integrated genome-wide association study (**GWAS**) and population genomics; and (3) resolve molecular mechanisms using tail-follicle transcriptomics, with emphasis on differential expression and genome-wide alternative splicing. This work establishes *PMEL* as a master switch for modular avian pigmentation and delivers actionable markers for ornamental and commercial chicken breeding.

## Materials and methods

### Ethics statement

All animal experiments were approved by the Animal Ethics Committee of Jiaying University (Approval No. JYDWLL2024-12). All experiments were conducted in compliance with the Guidelines for the Care and Use of Experimental Animals issued by Jiaying University. Standard protocols were followed to minimize animal stress and discomfort.

### Animals and phenotyping

Purebred Huaixiang roosters (BT phenotype, *n* = 5) and purebred Wuhua yellow hens (WT phenotype, *n* = 19) were obtained from the Fengdu Integrated Poultry Farm (Meizhou, China) and selected as parental (F_0_) lines. F_1_ progeny were generated by directional crossing (Huaixiang ♂ × Wuhua yellow ♀). A total of 131 F_1_ hybrid eggs were incubated, resulting in 97 F_1_ chicks. To maximize mapping resolution and genetic diversity, six F_1_ sires and twenty F_1_ dams, each derived from independent parental crosses, were intercrossed to produce the F_2_ population. From 198 incubated F_2_ eggs, 137 individuals survived to phenotyping age and comprised the final mapping population.

Eggs from both generations were incubated under standard poultry hatching protocols (temperature 37.8°C, relative humidity 65 %). After hatching, chicks were individually wing-banded for identification and reared in indoor net-floor pens (stocking density: 15 birds/m^2^) under a 16L:8D photoperiod. Birds were provided ad libitum access to water and a standard commercial corn–soybean-based diet formulated to meet the nutritional requirements of yellow-feathered chickens as per the Chinese feeding standard (NY/T 3645-2020). Tail-feather phenotype (BT or WT) was scored at 12 weeks of age by two independent observers; only individuals with unambiguous pure black or pure white tail feathers were retained for subsequent analyses.

### Genotyping and quality control

For genome-wide association mapping, 47 phenotypically extreme F_2_ roosters were selected: 32 WT and 15 BT. Selection was stratified across all 20 F_1_ dam families to minimize founder effects and kinship bias. At 12 weeks of age, blood samples were collected via wing vein puncture into EDTA-coated vacuum tubes and stored at −20°C. Genomic DNA was extracted from whole blood using a universal extraction kit (Guangzhou Magen Biotech, China) following the manufacturer’s protocol. DNA concentration and optical density were measured using a NanoDrop 2000 spectrophotometer (Thermo Fisher Scientific, USA), and purity was assessed by 1 % agarose gel electrophoresis.

DNA libraries with an ∼350-bp insert size were prepared using the MGIEasy PCR-Free DNA Library Prep Kit (MGI Tech Co., Ltd., Shenzhen, China). Paired-end sequencing (150 bp reads) was performed on a DNBSEQ-T7 platform (Beijing Berry Genomics, China), generating an average raw read coverage of 10× per sample.

Raw sequencing data were processed with Fastp v0.23.2 using default parameters (Chen, 2023). Reads were aligned to the chicken reference genome GRCg6a (assembly accession: GCA_000002315.5) with Burrows-Wheeler Aligner Maximal Exact Matches v0.7.17 (Li and Durbin, 2010). Post-alignment processing included coordinate sorting, merging, and duplicate marking/removal using Picard Tools v3.3.0. Variant calling was performed with Genome Analysis Toolkit HaplotypeCaller v4.2.0.0 (Mckenna et al., 2010) to obtain high-confidence single nucleotide polymorphisms (SNPs) and insertion–deletion (InDel). Initial variant filtering was conducted with GATK VariantFiltration using the following criteria: for SNPs, QD < 2.0 || MQ < 40.0 || FS > 60.0 || QUAL < 30.0 || SOR > 3.0 || MQRankSum < −12.5 || ReadPosRankSum < −8.0; and for InDels, QD < 2.0 || FS > 200.0 || SOR > 10.0 || MQRankSum < −12.5 || ReadPosRankSum < −8.0 || DP < 5. Additionally quality control included removing variants and samples with >10 % missing data, excluding variants with minor allele frequency < 0.01, eliminating variants violating Hardy–Weinberg equilibrium (*P* < 10E−6), and retaining only autosomal and Z chromosome biallelic variants for downstream analyses.

### Population structure and linkage disequilibrium analysis

To assess the genetic background of Wuhua yellow chickens, whole-genome sequencing data were compared with datasets from five published yellow-feathered chicken breeds possessing black tail feathers: Guangxi yellow, Hetian, Huaixiang, Huiyang bearded, and Jianghan chickens (*n* = 10 samples per breed) (Huang et al., 2020). Population structure was evaluated by principal component analysis (**PCA**) using VCF2PCACluster v1.41 (He et al., 2024) under the Normalized_IBS option. Linkage disequilibrium (**LD**) decay was analyzed using PopLDdecay v3.4 (Zhang et al., 2019) to assess genomic diversity patterns in the Wuhua yellow chicken population.

### Genome-wide association study analysis

A GWAS was conducted to analyze tail feather color traits using a linear mixed model implemented in Genome-wide Efficient Mixed Model Association v0.98.5 (Zhou and Stephens, 2012). The model included the first three principal components (**PC**s) and a kinship matrix as covariates to correct for population stratification and genetic relatedness. Significance thresholds were determined using Bonferroni correction based on the number of tested variants (N), with genome-wide significance set at *P* < 0.05/N and suggestive significance at *P* < 1/N. Variant associations were evaluated with the Wald test. GWAS results were visualized using the CMplot package (https://github.com/YinLiLin/CMplot) in R v4.4.2, and significant loci were functionally annotated using ANNOVAR v20240219 (Wang et al., 2010). LD block analysis was conducted using LDBlockShow v1.40 (Dong et al., 2021).

### Selection signature analysis

Selection signals were analyzed using Vcftools v0.1.16 (Danecek et al., 2011) with a 50-kb sliding window and 10-kb step size to calculate *F*_ST_ and nucleotide diversity (π) statistics across the 47 Wuhua yellow chickens and five additional chicken breeds from published data. Genomic regions under selection were identified as those falling within the top 1 % of both *F*_ST_ values and π-ratio distributions. Significant regions were annotated using Bedtools v2.27.1 (Quinlan and Hall, 2010). To improve reliability, candidate genes identified through GWAS, *F*_ST_, and π-ratio analyses were intersected using an R script. The resulting Ensembl IDs were mapped to corresponding genes within significant SNP regions via the Ensembl database.

### Transcriptomic analysis

Tail feather follicles were precisely dissected from the skin at the base of actively growing tail feathers. Eight biological replicates were collected: four WT and four BT male F_2_ individuals, all sampled at 8 weeks of age. Total RNA was extracted from tail follicle tissues using TRIzol reagent (Takara, Japan). RNA integrity and concentration were assessed by agarose gel electrophoresis and with a NanoDrop 2000 spectrophotometer (Thermo Fisher Scientific, USA). Qualified RNA samples were used to construct sequencing libraries, which were sequenced on the Illumina NovaSeq X Plus platform (GeneDenovo Biotechnology, China) to generate 150 bp paired-end reads. The raw sequencing data were processed using Fastp v0.23.2 (Chen, 2023) to remove adapter sequences and low-quality bases (quality threshold of 20), yielding clean reads for subsequent alignment and gene expression quantification. Clean reads were mapped to the chicken reference genome GRCg6a (assembly accession: GCA_000002315.5) using HISAT2 v2.1.0 with default parameters (Pertea et al., 2016). Gene expression was quantified with RSEM v1.3.1 (Li and Dewey, 2011) to calculate Transcripts Per Kilobase of exon model per Million mapped reads. Differential expression analysis was performed with DESeq2 v1.36.0 (Love et al., 2014) with significance thresholds of *P* < 0.05 and |log2(fold change)| ≥ 1. Gene Ontology (**GO**) and Kyoto Encyclopedia of Genes and Genomes (**KEGG**) pathway enrichment analyses were conducted using the KOBAS v3.0 online platform (Bu et al., 2021) (http://bioinfo.org/kobas) using an adjusted *P* < 0.05 to identify biological processes and pathways associated with white-tail feather coloration.

### Gene set enrichment analysis

Gene set enrichment analysis (**GSEA**) was performed using GSEA software v4.3.2 (Subramanian et al., 2005) with the Molecular Signatures Database (MSigDB v2023.1) to identify coordinated pathway-level changes between phenotypic groups. We analyzed GO Biological Processes. Whole-transcriptome expression data were ranked by signal-to-noise ratio, and enrichment scores were computed using 1,000 phenotype-based permutations. Gene sets with a false discovery rate (**FDR**) < 0.25 and a nominal *P* < 0.05, based on the normalized enrichment score (**NES**), were considered significantly enriched (Subramanian et al., 2005).

### Quantitative real-time PCR assay

Quantitative real-time PCR (**qPCR**) was performed to validate the RNA-seq results. High-quality total RNA was reverse-transcribed into cDNA using the HiScript IV All-in-One Ultra RT SuperMix for qPCR (Vazyme Biotech Co., Ltd, Nanjing, China) according to the manufacturer’s instructions. qPCR reactions were set up in a 10-μL volume using the Ipure SYBR Green Master Mix on a MA-6000 system (Suzhou Yairui Biological Technology Co., Ltd., Suzhou, China). Gene-specific primers (Table S1) were designed and validated for amplification efficiency and specificity. The thermocycling protocol was as follows: initial denaturation at 95°C for 10 min; 40 cycles of 95°C for 15 s and 61°C for 30 s. A melting curve analysis was performed at the end of each run to confirm the amplification of a single specific product. Gene expression levels were normalized to the endogenous control gene *GAPDH* and calculated using the 2^^−ΔΔCt^ method. All samples were run in technical triplicates.

### Alternative splicing analysis

AS events were identified and quantified using rMATS v4.3.0 (Wang et al., 2024). Differential splicing analysis between phenotypic groups was performed with aligned BAM files as input, using thresholds ≥10 junction reads and minimum effective length ≥ 50 bp. Events with FDR < 0.05 and |ΔPSI| > 0.10 (**PSI** = Percent Spliced In) were considered significant. This approach allows for the systematic detection of five major AS types: skipped exons (SE), mutually exclusive exons (MXE), retained introns (RI), and alternative 5ʹ and 3ʹ splice sites (A5SS and A3SS). Splicing patterns were visualized using sashimi plots generated with ggsashimi v1.1.5 (Garrido-Martin et al., 2018).

## Results

### Autosomal dominant inheritance of the white-tailed trait

The hybridization experiment was conducted by crossing purebred Wuhua yellow hens (WT phenotype) with purebred Huaixiang roosters (BT phenotype) (Fig. S1). All F_1_ progeny exhibited the WT trait with yellow body feathers, confirming the complete dominance of this trait. Six F_1_ sires were crossed with 20 F_1_ dams to generate the F_2_ generation, with phenotypic segregation data shown in Table 1. In the F_2_ population, the observed ratio of white- to black-tailed individuals was 3.28:1 (105:32), closely matching the expected 3:1 Mendelian ratio (χ^2^ = 0.20, *P* = 0.66). Sex distribution analysis showed 54 males to 51 females among WT chickens (χ^2^ = 0.09, *P* = 0.77) and 17 males to 15 females among BT chickens (χ^2^ = 0.13, *P* = 0.72), with an overall sex ratio of 71 males to 66 females (1.08:1, χ^2^ = 0.18, *P* = 0.67). These results collectively demonstrate autosomal inheritance without sex linkage and complete penetrance of the WT trait in Wuhua yellow chickens.

**Table 1:** Segregation of tail feather color traits in the F_2_ hybrid population.

F_1_		F_2_ (both sex)			F_2_ (White tail)			F_2_ (Black tail)		
Sire	Dams	White tail	Black tail	Ratio	Male	Female	Ratio	Male	Female	Ratio
A	5	25	9	2.78	16	9	1.78	3	6	0.50
B	4	22	3	7.33	12	10	1.20	1	2	0.50
C	4	17	3	5.67	10	7	1.43	1	2	0.50
D	3	16	7	2.29	7	9	0.78	4	3	1.33
E	2	13	3	4.33	4	9	0.44	2	1	2.00
F	2	12	7	1.71	5	7	0.71	6	1	6.00
Total	20	105	32	3.28	54	51	1.06	17	15	1.13

### Genomic landscape and population structure of the F_2_ cohort

After quality control, genomic data from 47 F_2_-generation samples yielded 12,451,489 SNPs and 1,373,882 InDels, with an average sequencing depth of 12.19× and mean genome coverage of 96.58 % (Table S2). When combined with 50 samples from five additional chicken breeds, the final dataset contained 12,775,271 SNPs, with an average sequencing depth of 10.73× and coverage of 96.41 % (Table S2).

**Fig. 1:**
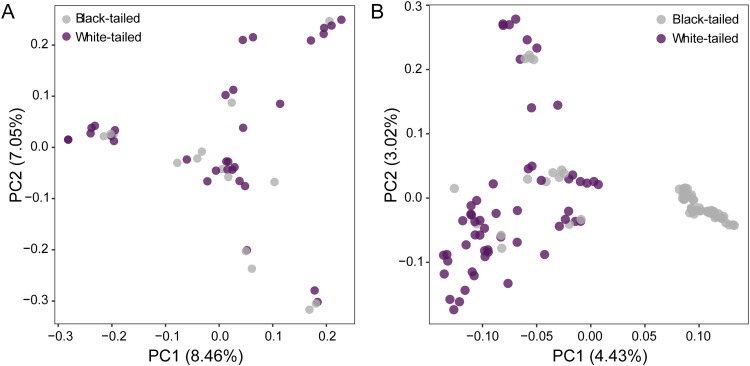
Principal component analysis (PCA) of tail feather phenotypes. (A) PCA of F_2_-generation individuals with white-tailed and black-tailed phenotypes. (B) Comparative PCA of white-tailed/black-tailed individuals (F_2_) with five additional yellow-feathered, black-tailed chicken breeds.

PCA of 47 F_2_ individuals with BT and WT phenotypes revealed no clear stratification between the two traits (Fig. 1A). However, expanded PCA including BT and WT individuals together with five BT chicken breeds (Huiyang bearded, Hetian, Jianghan, Huaixiang, and Guangxi yellow) showed clear genetic differentiation between WT and BT populations (Fig. 1B). Although WT and BT clustered separately, subtle variation within the WT group indicates population-level diversity in the white-tail feather trait.

### Genetic mapping and selection signatures at the PMEL locus

**Fig. 2:**
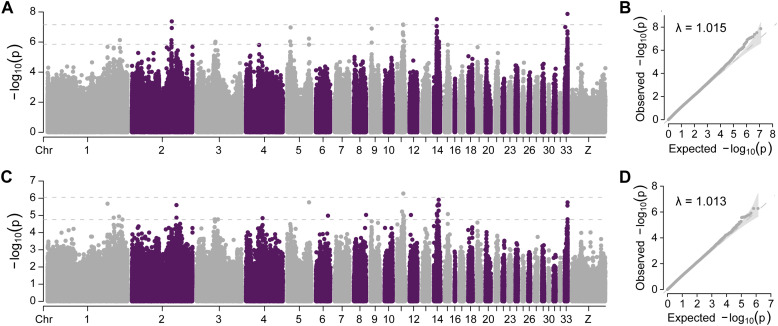
Genome-wide association study of F_2_ individuals. (A) Manhattan plots SNP-based associations. (B) Quantile-quantile plot of SNP analysis. (C) Manhattan plot of InDel associations. (D) Quantile-quantile plot of InDel analysis.

To identify the genetic determinants of the trait, GWAS of the WT trait was conducted using both SNPs and InDels (Fig. 2A,C). The quantile–quantile plots confirmed appropriate model fit, with most points aligning along the diagonal in lower quantiles and showing minimal inflation at higher quantiles (Fig. 2B,D). The analysis identified 56 significant SNPs (*P* < 2.88E−7) and 42 InDels (*P* < 1.77E−5), each annotated to 39 Ensembl genes. After merging results, 57 unique genes were identified across chromosomes 1–6, 8, 9, 11, 12, 14, 15, and 33 (Table S3,S4). The strongest association was detected at *PMEL* locus on chromosome 33 (top SNP: *P* = 1.35E−8) (Table S3).

**Fig. 3:**
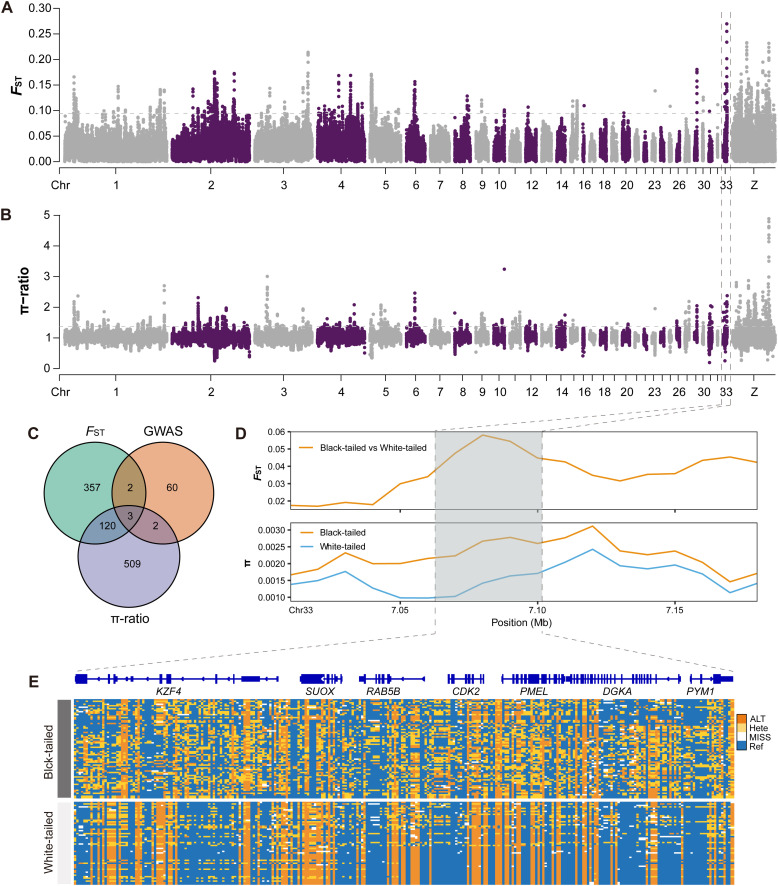
Genome-wide selection signal analysis in F_2_ individuals. (A) Manhattan plots of *F*_ST_ values. (B) Manhattan plots of π-ratio values. (C) Venn diagram of overlapping candidate genes from GWAS and selection analyses. (D) Selective sweeps specific to the white-tailed phenotype. (E) Genotype pattern of SNPs within selected regions.

To support the GWAS findings, the *F*_ST_ analysis identified 790 putative selected regions (*F*_ST_ > 0.093) containing 482 Ensembl genes, while π-ratio analysis detected 788 regions (π-ratio > 1.373) with 635 Ensembl genes (Fig. 3A,B; Table S5,S6). Venn diagram analysis integrating GWAS, *F*_ST_, and π-ratio results revealed three overlapping genes (Fig. 3C), which mapped to *IKZF4, PMEL*, and *PYM1*, all located on chromosome 33. These were designated as positively selected genes (**PSG**s). Selection signals and haplotype analyses demonstrated strong selective sweeps across these loci (Fig. 3D,E), with *PMEL*, a key melanogenesis gene, exhibiting the strongest signal (*F*_ST_ = 0.271, π-ratio = 2.203). Among the three PSGs, seven significant GWAS SNPs within 20 kb of each locus were analyzed for LD . However, only one SNP formed an LD block in the *PYM1* region (Fig. S2).

### Transcriptomic profiling reveals downregulation of melanogenesis pathways

**Fig. 4:**
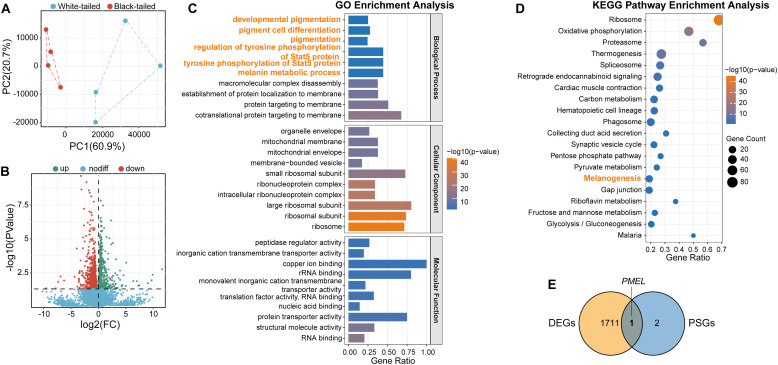
Transcriptomic profiling of F_2_ hybrids with contrasting tail pigmentation phenotypes. (A) Principal component analysis (PCA) segregating WT and BT individuals. (B) Volcano plot of differentially expressed genes (DEGs). Upregulated (up), downregulated (down), and non-differential (nodiff) transcripts are shown in green, red, and blue, respectively. (C) Gene Ontology (GO) enrichment analysis across biological process, cellular component, and molecular function categories. (D) Kyoto Encyclopedia of Genes and Genomes (KEGG) pathways enrichment. Bubble size represents gene count; color intensity reflects statistical significance. (E) Integration of genomic selection signals and transcriptomic differences. The Venn diagram highlights overlap between positively selected genes (PSGs) and tail phenotype-associated DEGs, prioritizing *PMEL* as a key candidate gene.

Having genetically localized the candidate region, we next investigated the underlying transcriptional changes. Transcriptome sequencing of eight samples yielded high-quality data (average: 46.66 million clean reads per sample; mapping ratio: 93.70 %; Q30 score > 93.60 %; Table S7). PCA confirmed distinct transcriptomic profiles between WT and BT groups (Fig. 4A). Differential expression analysis identified 1,712 differentially expressed genes (**DEG**s), including 918 upregulated and 794 downregulated in WT compared with those in BT (Fig. 4B; Table S8). Functional enrichment revealed significant overrepresentation of pigmentation-related pathways, involving key regulators (*TYR, TYRP1, KIT, SOX10, OCA2, EDN3*, and *PMEL*), with specific enrichment of the KEGG melanogenesis pathway (ko04916) (Fig. 4C,D; Table S9,S10). The coordinated downregulation of this core pathway was striking, with tyrosinase (TYR; log_2_FC = −0.99, FDR = 9.93E−4) and especially *PMEL* showing the most pronounced suppression (log_2_FC = −2.18, FDR = 5.58E−06), provides a direct molecular explanation for the failure to produce eumelanin in white-tail feathers. Integrating DEGs with PSGs prioritized *PMEL* as common candidate gene (Fig. 4E), highlighting its role in melanosome maturation and feather pigmentation.

### Integrative validation of PMEL as the candidate gene

**Fig. 5:**
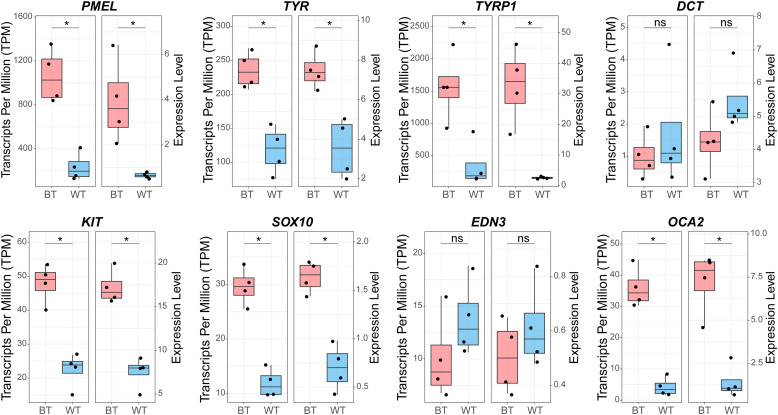
Expression profiles of candidate genes in tail feather follicles. BT denotes F_2_ hybrids with black tail feathers, and WT indicates F_2_ hybrids with white-tailed trait. Left panels show RNA-seq data (Transcripts Per Million, TPM), and the right panels show quantitative real-time PCR (qPCR) data (expression level). Statistical significance was determined using Wilcoxon rank-sum tests with Bonferroni correction (*P < 0.05).

Transcriptomic analysis revealed significant downregulation of *PMEL, TYR, TYRP1, KIT, SOX10*, and *OCA2* in WT compared with that in BT chickens, along with upregulation of *DCT* and *EDN3* in WT follicles (Table S8). Statistical testing confirmed significant inter-group differences for the downregulated genes (*PMEL, TYR, TYRP1, KIT, SOX10*, and *OCA2; P* < 0.05), but not for *DCT* and *EDN3* (Fig. 5). qPCR validation supported the RNA-seq results (Fig. 5). These findings further support *PMEL* as a strong candidate gene underlying pigmentation differences.

**Fig. 6:**
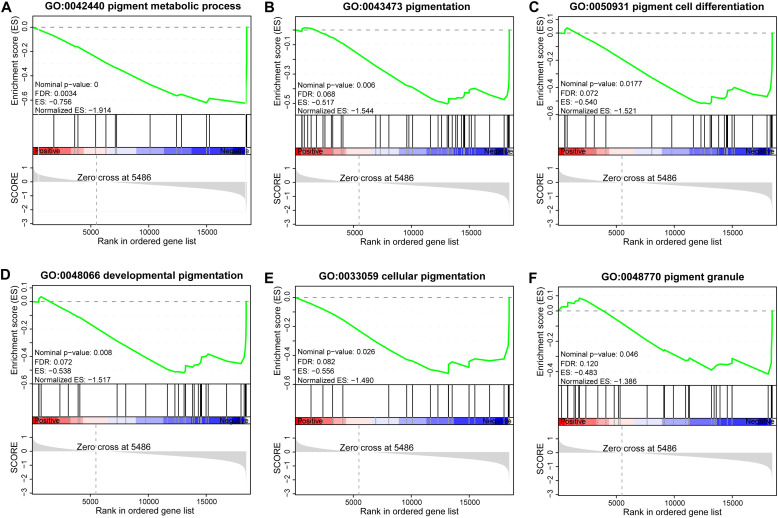
Gene Set Enrichment Analysis (GSEA) of pigmentation-related pathways in black-tailed (BT) versus white-tailed (WT) chickens. The enrichment score (ES) trajectory (green line) shows gene set distribution within a ranked expression list (left: WT-enriched; right: BT-enriched). Positive ES peaks denote enrichment in WT, negative troughs indicate enrichment in BT. Vertical dashed lines mark zero-crossing points; black ticks denote leading-edge genes.

To complement the differential expression analysis, GSEA was performed to assess coordinated, pathway-level changes between WT and BT chickens. Six significantly enriched GO terms related to pigmentation regulation were identified, four of which included *PMEL* in their core enrichment gene sets (Fig. 6; Table S11). Notably, the pigment metabolic process (GO:0042440) exhibited a strongly negative value (NES = –1.914, nominal *P* = 0, FDR = 0.003), indicating coordinated suppression of this pathway in the WT group compared to the BT group (Fig. 6A). Core enrichment genes driving this signature included *TYR, TYRP1, PMEL*, and *OCA2*. Collectively, these genomic–transcriptomic findings confirm *PMEL* as a key regulator of pigment synthesis and its significant suppression in the WT phenotype.

### Alternative splicing patterns at the PMEL locus

**Fig. 7:**
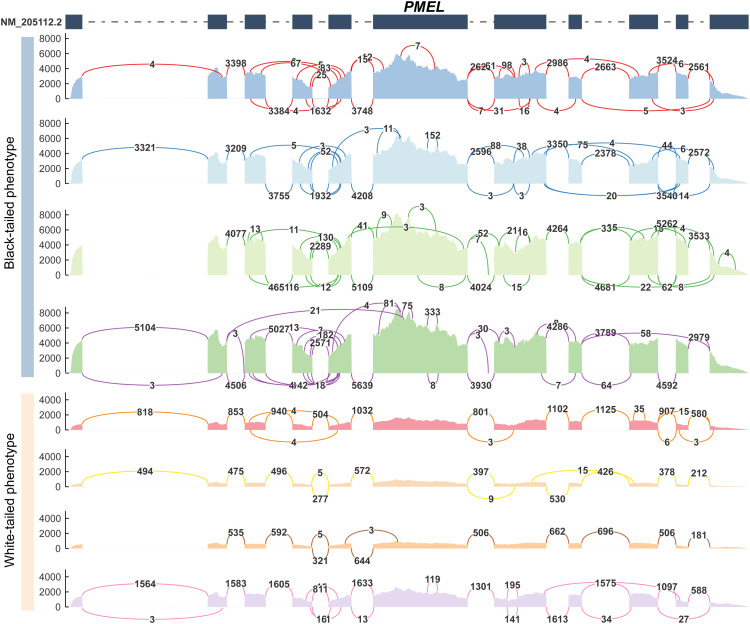
Alternative splicing of the *PMEL* locus in black-tailed (BT) versus white-tailed (WT) chickens. Sashimi plots display exon connectivity patterns. Arcs represent splicing events, with numbers denoting supporting read counts. Upper panels: BT individuals show complex splicing with frequent long-range arcs (spanning ≥2 exons). Lower panels: WT individuals exhibit reduced splicing dominated by short-range connections.

Having established *PMEL*’s central role through its significant transcriptional suppression, we next investigated potential co-occurring post-transcriptional regulation. Genome-wide analysis identified widespread phenotypic divergence in AS, with 2,764 events associated with tail color that encompassed all five major AS types (SE, RI, A5SS, A3SS, and MXE), indicating a systemic regulatory difference (Table S12). At the *PMEL* locus itself, a striking pattern of splicing simplification emerged in WT individuals (Table S13). Among the five detected events, two showed reduced exon inclusion or usage in WT chickens (negative ΔPSI). This overall trend toward reduced variation was visually confirmed by exon connectivity analysis: BT individuals exhibited substantially greater splicing complexity with frequent long-range splicing arcs spanning multiple exons, indicative of broad isoform diversification (Fig. 7), whereas WT individuals showed simplified architecture dominated by short-range connections. Collectively, these findings reveal a multi-layered regulatory mechanism at the *PMEL* locus that integrates profound transcriptional suppression with reduced splicing complexity to ensure the tail-specific depigmentation phenotype.

## Discussion

Feather pigmentation patterns are governed by complex genetic mechanisms that often exhibit region-specific regulation in avian species (Ng and Li, 2018). The WT trait in Wuhua yellow chickens is not only of economic importance but also provides a useful model for studying localized pigment patterning. Here, we combined hybridization experiments, GWAS, selection signature analyses, functional enrichment, and gene expression analysis to elucidate the genetic architecture of this trait. Our results demonstrate that tail feather color is regulated independently of body plumage, with *PMEL* identified as a key candidate gene underlying the WT phenotype. This integrated approach moves beyond traditional single-gene mutation studies by revealing a multi-layered regulatory mechanism that provides a more comprehensive explanation for this spatially restricted trait.

### Autosomal dominant inheritance decouples tail depigmentation from body plumage

Feather color inheritance often follows Mendelian patterns (Ng and Li, 2018). Our data revealed an autosomal dominant mode for WT feathers, supported by (i) complete dominance in F_1_ progeny (100 % WT), (ii) a 3:1 segregation ratio in the F_2_ generation (105 WT: 32 BT, χ^2^ = 0.20, *P* = 0.66), and (iii) balanced sex ratios (WT: 54♂/51♀; BT: 17♂/15♀). Together, these results confirm that WT feathers in Wuhua yellow chickens represent a simple autosomal dominant trait that is independent of body plumage.

This genetic decoupling is consistent with previous studies on tail feather color inheritance in chickens (Nie et al., 2021; Zheng et al., 2024). Nie et al. (2021) reported autosomal inheritance of tail feather color in dwarf chickens, though involving a different locus. Zheng et al. (2024) demonstrated that in Huiyang bearded chickens, tail feather pigmentation is regulated independently from body plumage, with the *Dominant White* locus affecting tail color and the *Silver* locus controlling body plumage. Comparable, region-specific inheritance patterns are observed across vertebrates, such as dorsal–ventral pigmentation differences in mice (Vrieling et al., 1994) and breast-specific plumage variation in geese (Yang et al., 2024), where pigment traits segregate independently from overall body coloration. Such modular control highlights the evolutionary advantage of dedicated genetic switches that sculpt regional phenotypes without perturbing global coloration.

### PMEL as the primary candidate gene of tail depigmentation

Integrated genomic analyses pinpointed a major locus on chromosome 33 harboring *PMEL* as the sole credible causal gene. *PMEL* encodes a melanosomal matrix protein essential for eumelanin synthesis, and mutations in *PMEL* have been linked to plumage depigmentation in diverse avian species (Andersson et al., 2020). The spatial restriction of *PMEL*’s effect on tail feathers parallels simillar mechanisms observed in other species. In felids, for instance, *Taqpep*-dependent pre-patterns and *EDN3*-mediated melanocyte regulation create localized tabby markings (Kaelin et al., 2021). Similarly, the white tiger retains its stripe patterning despite *SLC45A2*-mediated pheomelanin suppression (Xu et al., 2013). These cross-species parallels underscore the complexity of region-specific pigment regulation. Our findings extend previous research in domestic ducks and chickens, where *PMEL* mutations reduce eumelanin production and result in white plumage (Kerje et al., 2004; Wang et al., 2025b). Cross-species comparisons further corroborate this relationship, as evidenced by higher *PMEL* expression in dark-feathered Silkie chickens (Peng, 2018) and lower expression in L strain quail compared to WE morphs (Ishishita et al., 2018). The identification of *PMEL* as the driver of localized pigmentation aligns with broader vertebrate patterns, where genes like *SLC45A2* and *NDP* exert context-dependent effects on pigment patterning (Xu et al., 2013; Vickrey et al., 2018). Moreover, functional enrichment analysis further supported *PMEL*’s central role through its association with melanosome organization pathways.

Transcriptomic analysis provided mechanistic insights into this process. We detected significant downregulation of *PMEL* expression in WT chickens relative to BT at 8 weeks of age (log_2_FC = –2.18, *P* < 0.05; Fig. 5). This downregulation was validated independently by qPCR (Fig. 5). This result is physiologically consistent with *PMEL*’s role as a structural determinant of melanosome maturation and eumelanin deposition. Notably, this expression difference at eight weeks contrasts with reports of higher *PMEL* expression in white-feathered quail embryos compared to brown-feathered ones (Yuan et al., 2023), highlighting potential stage-specific regulation. Similar temporal dynamics have been documented in geese, where *EDNRB2*-associated melanocyte defects manifest only during defined developmental windows (Yang et al., 2024). Collectively, these results indicate that pigment-related genes, including *PMEL*, exhibit phase-dependent expression profiles during feather development (Manceau et al., 2011; Lin et al., 2013; Zheng et al., 2024). Thus, the significant downregulation detected at eight weeks provides a molecular correlate for the WT phenotype while suggesting that earlier stages may involve distinct regulatory dynamics.

An important contrast emerges between our chromosome 33/*PMEL* locus and Nie et al.’s (2021) chromosome 24/*MCAM* findings, highlighting breed-specific mechanisms of tail feather pigmentation. Similarly, Zheng et al. (2023) identified *ASIP* as the key regulator in yellow-bodied chickens with BT traits, underscoring the genetic complexity of feather pigmentation. Notably, our transcriptomic data revealed no significant expression differences in *MCAM* or *ASIP* between WT and BT individuals (table S8). This result demonstrates that divergent genetic pathways can produce the same regional phenotype, an effect potentially driven by distinct selection pressures or redundant regulatory networks. The identification of *PMEL* as the causative locus in this specific genetic background not only clarifies the genetic architecture of a commercially valuable trait but also highlights a distinct molecular target.

The regulatory haplotypes and transcriptional suppression mechanism we uncovered provide a potential path for application. They enable marker-assisted selection for the white-tail trait, allowing breeders to accurately fix this phenotype without affecting yellow body plumage, thereby enhancing market appeal and breed purity. This gene-centric approach has proven effective, as shown by *PMEL*-based selection in other chicken breeds (Chai et al., 2023). Integration with other known loci, such as those controlling feather color in ducks (Sun et al., 2023), could further enable comprehensive molecular breeding strategies. This direction aligns with current industry trends in genetic trait improvement (Wang et al., 2025a), promoting more efficient and sustainable poultry production.

### Alternative splicing dysregulation underlies PMEL-mediated tail-specific depigmentation

Beyond transcriptional regulation, AS serves as a crucial post-transcriptional mechanism that expands proteomic diversity and fine-tunes gene function in spatiotemporal contexts (Wright et al., 2022; Choquet et al., 2025). Our transcriptomic analysis provided two key lines of evidence for its role in this trait: a genome-wide identification of 2,764 phenotype-associated splicing events, and a noticeable reduction in splicing complexity specifically at the *PMEL* locus in WT chickens (Fig. 7). This suggests a novel layer of regulation in avian pigmentation genetics, consistent with findings that AS contributes significantly to phenotypic diversity and evolutionary innovation across species (Choquet et al., 2025).

The observed divergence suggests that the splicing landscape in WT chickens is shifted towards a less complex architecture. We therefore hypothesize that the core mechanism driving localized tail depigmentation involves the combined effects of profound transcriptional suppression and a subtle yet coordinated dampening of splicing diversity at the *PMEL* locus. This multi-layered regulatory mechanism may collectively limit the production of the full spectrum of functional PMEL isoforms necessary for proper melanosome maturation specifically within tail feather follicles. The capacity for splicing modulation to produce regional effects is well established, as demonstrated by the region-restricted AS of *ASIP* governing dorsal–ventral pigmentation in mice (Manceau et al., 2011).

From a functional and evolutionary perspective, the ability to fine-tune pigmentation through AS, alongside transcriptional control, offers a flexible mechanism for adaptation and diversification (Bhuiyan et al., 2018). This regulatory versatility could allow for precise adjustment of color patterns in response to selective pressures without fundamentally altering the core coding sequences of melanogenic genes. The existence of breed-specific genetic mechanisms (e.g., *PMEL* vs. *MCAM*/*ASIP*) (Nie et al., 2021; Zheng et al., 2024) further highlights the evolutionary plasticity of pigmentation regulation. It demonstrates that distinct molecular pathways, operating at different regulatory levels, can converge on similar phenotypic outcomes.

### Developmental dynamics, regulatory networks, and future directions

Our transcriptomic analysis revealed that several key melanogenesis genes are significantly downregulated in WT tail follicles compared with those in BT. These genes include *TYR, TYRP1, DCT, KIT, SOX10*, and *OCA2* (Fig. 5). However, these genes showed no strong selection signatures in our GWAS or population genomic scans. This discrepancy likely reflects the polygenic nature of pigmentation regulation (Andersson et al., 2020). Genes involved in melanin synthesis, transport, and melanocyte development often function within tightly co-regulated networks (Baralle and Giudice, 2017; Ule and Blencowe, 2019), where coordinated expression is modulated by master regulators such as *MITF,* or by localized signals such as *EDN3*, rather than by strong selection on individual coding variants. The prominence of *PMEL* as a candidate gene likely stems from its structural role in eumelanosome formation, which makes it a bottleneck in the pathway. Thus, the downregulation of other pigment genes in WT individuals may represent either a downstream consequence of defective melanosome biogenesis caused by *PMEL* deficiency, or coordinated suppression within the pigment gene network specific to tail follicles.

The non-significant trend in *DCT* and *EDN3* expression differences between WT and BT groups further suggests developmental-stage specificity. Notably, *EDN3*-mediated melanocyte migration typically precedes *PMEL*-dependent maturation (Kaelin et al., 2021). Our sampling at 8 weeks may have captured a stage when melanocyte migration and early differentiation signals mediated by *EDN3* had subsided, whereas the structural role of *PMEL* remained critical. This would explain the significant downregulation of *PMEL* but not *EDN3*. This interpretation aligns with quail studies showing embryonic peaks for some pigment genes (Yuan et al., 2023). Future studies should therefore examine embryonic and early post-hatching stages to resolve the temporal dynamics of gene expression and network activation during tail feather pigmentation.

While our integrated approach provides compelling evidence for *PMEL*’s role in tail-specific depigmentation, several limitations of this study warrant consideration. First, the moderate sample sizes for GWAS (*n* = 47) and transcriptome (*n* = 8) analyses, though adequate for identifying the major locus, limit the resolution for fine-mapping and for pinpointing the precise causal mutation(s) within the *PME*L region. Second, our findings are primarily based on a specific F_2_ cross; the lack of validation in broader, independent chicken populations means that the general applicability of these specific genetic variants remains to be fully confirmed. Third, our analysis revealed a global trend of reduced splicing complexity and a coordinated pattern at the *PMEL* locus. Still, the moderate depth of the RNA-seq data limited our ability to accurately quantify the abundance of specific, low-expression transcript isoforms. Future studies should employ long-read sequencing to resolve the precise structure of phenotype-associated isoforms and combine this with spatiotemporal transcriptomic data and co-expression network analysis to dissect the regulatory hierarchy. Furthermore, experimental validation to determine the functional impact of specific splicing events on PMEL protein localization and melanosome biogenesis will be essential to confirm their causal role in the depigmentation phenotype.

## Conclusions

This study establishes that the autosomal dominant inheritance of the white-tail trait in Wuhua yellow chickens is primarily governed by the *PMEL* gene. We demonstrate that this phenotype arises through breed-specific transcriptional suppression and reduced splicing complexity at the *PMEL* locus, which disrupts melanogenesis in a spatially restricted manner, independent of the yellow body plumage. This localized regulatory mechanism provides a novel genetic model for understanding regional pigment patterning in birds. The robust association between *PMEL* genotypes and the dominant white-tail phenotype offers a clear genetic basis for trait selection in breeding programs. Looking forward, resolving the exact PMEL isoform structures through long-read sequencing and functionally validating their effects will be crucial to elucidate this post-transcriptional regulatory mechanism. Ultimately, our findings provide a new molecular framework for understanding the spatiotemporal control of pigmentation and open avenues for applying transcript isoform regulation in precision poultry breeding.

## Funding

This work was supported by the Peak Talent Program of Jiaying University (2022RC45), the Key Discipline Construction Project of Guangdong Provincial Department of Education (2022ZDJS088), and the Scientific Research Innovation Team Project of Jiaying University (2021JYUTDS04).

## CRediT authorship contribution statement

**Xunhe Huang:** Writing – review & editing, Writing – original draft, Visualization, Supervision, Resources, Project administration, Methodology, Investigation, Funding acquisition, Formal analysis, Data curation, Conceptualization. **Luheng Zhang:** Writing – review & editing, Validation, Resources, Data curation. **Zhipeng Zhong:** Writing – review & editing, Validation, Resources, Data curation. **Zhuoxian Weng:** Writing – review & editing, Resources. **Yongjie Xu:** Writing – review & editing, Resources. **Jintian Wen:** Writing – review & editing, Visualization, Methodology. **Cheng Ma:** Writing – review & editing, Methodology. **Bingwang Du:** Writing – review & editing, Resources.

## Disclosures

The authors declare that they have no known competing financial interests or personal relationships that could have appeared to influence the work reported in this paper.

## Appendix Supplementary materials

Image, application 1

Image, application 2

Image, application 3

Image, application 4

Image, application 5

Image, application 6

Image, application 7

Image, application 8

Image, application 9

Image, application 10

Image, application 11

Image, application 12

Image, application 13

Image, application 14

Image, application 15

## Footnotes

The sequencing data have been deposited in the NCBI Sequence Read Archive under the BioProject accession number PRJNA1290005 and PRJNA1313834. Supplementary material associated with this article can be found, in the online version, at doi:10.1016/j.psj.2025.106127.

## References

[bib0001] Andersson L., Bed'Hom B., Chuong C., Inaba M., Okimoto R., Tixier-Boichard M., Aggrey S.E., Zhou H., Tixier-Boichard M., Rhoads D.D. (2020). Advances in Poultry Genetics and Genomics.

[bib0002] Anello M., Daverio M.S., Rodríguez S.S., Romero S.R., Renieri C., Vidal Rioja L., Di Rocco F. (2022). The ASIP gene in the llama (*Lama glama*): alternative transcripts, expression and relation with color phenotypes. Gene.

[bib0003] Baralle F.E., Giudice J. (2017). Alternative splicing as a regulator of development and tissue identity. Nat. Rev. Mol. Cell Biol..

[bib0004] Bhuiyan S.A., Ly S., Phan M., Huntington B., Hogan E., Liu C.C., Liu J., Pavlidis P. (2018). Systematic evaluation of isoform function in literature reports of alternative splicing. BMC Genom..

[bib0005] Bu D., Luo H., Huo P., Wang Z., Zhang S., He Z., Wu Y., Zhao L., Liu J., Guo J., Fang S., Cao W., Yi L., Zhao Y., Kong L. (2021). KOBAS-i: intelligent prioritization and exploratory visualization of biological functions for gene enrichment analysis. Nucleic Acids Res..

[bib0006] Chai X., Gong J., Cui F., Kang Z., Wu Y., Zhou M., Sun X., Xu J. (2023). Molecular marker-assisted selection of green shank and white feather traits in chickens. Guangdong Agric. Sci..

[bib0007] Chen S. (2023). Ultrafast one-pass FASTQ data preprocessing, quality control, and deduplication using fastp. Imeta.

[bib0008] Choquet K., Patop I.L., Churchman L.S. (2025). The regulation and function of post-transcriptional RNA splicing. Nat. Rev. Genet..

[bib0009] Cooke T.F., Wu P., Jiang T.X., Xie K.T., Kuo J., Doctorov E., Zehnder A., Khosla C., Chuong C.M., Bustamante C.D. (2017). Genetic mapping and biochemical basis of yellow feather pigmentation in budgerigars. Cell.

[bib0010] D’Alba L., Shawkey M.D. (2019). Melanosomes: biogenesis, properties, and evolution of an ancient organelle. Physiol. Rev..

[bib0011] Danecek P., Auton A., Abecasis G., Albers C.A., Banks E., DePristo M.A., Handsaker R.E., Lunter G., Marth G.T., Sherry S.T., McVean G., Durbin R. (2011). The variant call format and VCFtools. Bioinformatics.

[bib0012] Dong S.S., Ji J.J., Zhang C., Guo Y., Yang T.L. (2021). LDBlockShow: a fast and convenient tool for visualizing linkage disequilibrium and haplotype blocks based on variant call format files. Brief Bioinform..

[bib0013] Elkin J., Martin A., Courtier-Orgogozo V., Santos M. (2023). Analysis of the genetic loci of pigment pattern evolution in vertebrates. Biol. Rev..

[bib0014] Garrido-Martin D., Palumbo E., Guigo R., Breschi A. (2018). ggsashimi: sashimi plot revised for browser- and annotation-independent splicing visualization. PLoS Comput. Biol..

[bib0015] He W., Xu L., Wang J., Yue Z., Jing Y., Tai S., Yang J., Fang X. (2024). VCF2PCACluster: a simple, fast and memory-efficient tool for principal component analysis of tens of millions of SNPs. BMC Bioinform..

[bib0016] Horecka B., Wojciechowski W., Drabik K., Wengerska K., Batkowska J. (2024). Characterization of the coding sequence of the *MC1R* (Melanocortin 1 Receptor) gene of Ayam Cemani black chickens. Animals (Basel).

[bib0017] Huang X., Otecko N.O., Peng M., Weng Z., Li W., Chen J., Zhong M., Zhong F., Jin S., Geng Z., Luo W., He D., Ma C., Han J., Ommeh S.C., Zhang Y., Zhang X., Du B. (2020). Genome-wide genetic structure and selection signatures for color in 10 traditional Chinese yellow-feathered chicken breeds. BMC Genom..

[bib0018] Ishishita S., Takahashi M., Yamaguchi K., Kinoshita K., Nakano M., Nunome M., Kitahara S., Tatsumoto S., Go Y., Shigenobu S., Matsuda Y. (2018). Nonsense mutation in *PMEL* is associated with yellowish plumage colour phenotype in Japanese quail. Sci. Rep..

[bib0019] Kaelin C.B., McGowan K..A., Barsh G.S. (2021). Developmental genetics of color pattern establishment in cats. Nat. Commun..

[bib0020] Kawakami A., Fisher D.E. (2017). The master role of microphthalmia-associated transcription factor in melanocyte and melanoma biology. Lab. Invest..

[bib0021] Kerje S., Sharma P., Gunnarsson U., Kim H., Bagchi S., Fredriksson R., Schutz K., Jensen P., von Heijne G., Okimoto R., Andersson L. (2004). The dominant white, Dun and smoky color variants in chicken are associated with insertion/deletion polymorphisms in the *PMEL17* gene. Genetics.

[bib0022] Kratochwil C.F., Mallarino R. (2023). Mechanisms underlying the formation and evolution of vertebrate color patterns. Annu. Rev. Genet..

[bib0023] Li B., Dewey C.N. (2011). RSEM: accurate transcript quantification from RNA-seq data with or without a reference genome. BMC Bioinform..

[bib0024] Li H., Durbin R. (2010). Fast and accurate long-read alignment with Burrows-Wheeler transform. Bioinformatics.

[bib0025] Lin S.J., Foley J.., Jiang T.X., Yeh C.Y., Wu P., Foley A., Yen C.M., Huang Y.C., Cheng H.C., Chen C.F., Reeder B., Jee S.H., Widelitz R.B., Chuong C.M. (2013). Topology of feather melanocyte progenitor niche allows complex pigment patterns to emerge. Science.

[bib0026] Love M.I., Huber W.., Anders S. (2014). Moderated estimation of fold change and dispersion for RNA-seq data with DESeq2. Genome Biol..

[bib0027] Manceau M., Domingues V.S., Mallarino R., Hoekstra H.E. (2011). The developmental role of Agouti in color pattern evolution. Science.

[bib0028] McKenna A., Hanna M., Banks E., Sivachenko A., Cibulskis K., Kernytsky A., Garimella K., Altshuler D., Gabriel S., Daly M., DePristo M.A. (2010). The Genome Analysis Toolkit: a MapReduce framework for analyzing next-generation DNA sequencing data. Genome Res..

[bib0029] Ng C.S., Li W. (2018). Genetic and molecular basis of feather diversity in birds. Genome Biol. Evol..

[bib0030] Nie C., Qu L., Li X., Jiang Z., Wang K., Li H., Wang H., Qu C., Qu L., Ning Z. (2021). Genomic regions related to white/black tail feather color in dwarf chickens identified using a genome-wide association study. Front. Genet..

[bib0031] Peng C. (2018).

[bib0032] Pertea M., Kim D., Pertea G.M., Leek J.T., Salzberg S.L. (2016). Transcript-level expression analysis of RNA-seq experiments with HISAT, StringTie and Ballgown. Nat. Protoc..

[bib0033] Popadic A., Tsitlakidou D. (2021). Regional patterning and regulation of melanin pigmentation in insects. Curr. Opin. Genet. Dev..

[bib0034] Price-Waldman R., Stoddard M.C. (2021). Avian coloration genetics: recent advances and emerging questions. J. Hered..

[bib0035] Quinlan A.R., Hall I.M. (2010). BEDTools: a flexible suite of utilities for comparing genomic features. Bioinformatics.

[bib0036] Subramanian A., Tamayo P., Mootha V.K., Mukherjee S., Ebert B.L., Gillette M.A., Paulovich A., Pomeroy S.L., Golub T.R., Lander E.S., Mesirov J.P. (2005). Gene set enrichment analysis: a knowledge-based approach for interpreting genome-wide expression profiles. Proc. Natl. Acad. Sci. U.S.A..

[bib0037] Sun Y., Wu Q., Lin R., Chen H., Zhang M., Jiang B., Wang Y., Xue P., Gan Q., Shen Y., Chen F., Liu J., Zhou C., Lan S., Pan H., Deng F., Yue W., Lu L., Jiang X., Li Y. (2023). Genome-wide association study for the primary feather color trait in a native Chinese duck. Front. Genet..

[bib0038] Ule J., Blencowe B.J. (2019). Alternative splicing regulatory networks: functions, mechanisms, and evolution. Mol. Cell.

[bib0039] Vickrey A.I., Kronenberg Z., Mackey E., Bohlender R.J., Maclary E.T., Maynez R., Osborne E.J., Johnson K.P., Huff C.D., Yandell M., Shapiro M.D. (2018). Introgression of regulatory alleles and a missense coding mutation drive plumage pattern diversity in the rock pigeon. Elife.

[bib0040] Vrieling H., Duhl D.M., Millar S.E., Miller K.A., Barsh G.S. (1994). Differences in dorsal and ventral pigmentation result from regional expression of the mouse agouti gene. Proc. Natl. Acad. Sci. U.S.A..

[bib0041] Wang K., Li M., Hakonarson H. (2010). ANNOVAR: functional annotation of genetic variants from high-throughput sequencing data. Nucleic Acids Res..

[bib0042] Wang J., Zhu S., Xiong X., Qiu M., Zhang Z., Hu C., Yang L., Peng H., Song X., Chen J., Xia B., Xiong Z., Du L., Yu C., Yang C. (2025). Research progress on the molecular mechanism of poultry feather follicle development. Curr. Issues Mol. Biol..

[bib0043] Wang Y., Xie Z., Kutschera E., Adams J.I., Kadash-Edmondson K.E., Xing Y. (2024). rMATS-turbo: an efficient and flexible computational tool for alternative splicing analysis of large-scale RNA-seq data. Nat. Protoc..

[bib0044] Wang Z., Guo Z., Liu H., Liu T., Liu D., Yu S., Tang H., Zhang H., Mou Q., Zhang B., Cao J., Schroyen M., Hou S., Zhou Z. (2025). A high-quality assembly revealing the *PMEL* gene for the unique plumage phenotype in Liancheng ducks. Gigascience.

[bib0045] Wen J., Shao P., Chen Y., Wang L., Lv X., Yang W., Jia Y., Jiang Z., Zhu B., Qu L. (2021). Genomic scan revealed *KIT* gene underlying white/gray plumage color in Chinese domestic geese. Anim. Genet..

[bib0046] Weng Z., Xu Y., Li W., Chen J., Zhong M., Zhong F., Du B., Zhang B., Huang X. (2020). Genomic variations and signatures of selection in Wuhua yellow chicken. PLoS One.

[bib0047] Wright C.J., Smith C.W.J., Jiggins C.D. (2022). Alternative splicing as a source of phenotypic diversity. Nat. Rev. Genet..

[bib0048] Xu X., Dong G., Hu X., Miao L., Zhang X., Zhang D., Yang H., Zhang T., Zou Z., Zhang T., Zhuang Y., Bhak J., Cho Y.S., Dai W., Jiang T., Xie C., Li R., Luo S. (2013). The genetic basis of white tigers. Curr. Biol..

[bib0049] Yang Y., Wang H., Liu Y., Zhai S., Liu H., He D. (2024). A novel codominant plumage color pattern of white breast patches in WugangTong geese was controlled by EDNRB2. Poult. Sci..

[bib0050] Yuan Z., Zhang X., Pang Y., Qi Y. (2023). Association analysis of PMEL gene expression and single nucleotide polymorphism with plumage color in quail. Anim. Biotechnol..

[bib0051] Zhang C., Dong S.S., Xu J.Y., He W.M., Yang T.L. (2019). PopLDdecay: a fast and effective tool for linkage disequilibrium decay analysis based on variant call format files. Bioinformatics.

[bib0052] Zhang X., Zhu T., Wang L., Lv X., Yang W., Qu C., Li H., Wang H., Ning Z., Qu L. (2023). Genome-wide association study reveals the genetic basis of duck plumage colors. Genes (Basel).

[bib0053] Zheng X., Chen J., Nie R., Miao H., Chen Z., He J., Xie Y., Zhang H. (2024). Differential expression of *ASIP* transcripts reveals genetic mechanism underpinning black-tail independence from body plumage in yellow-bodied chickens. Anim. Genet..

[bib0054] Zhou X., Stephens M. (2012). Genome-wide efficient mixed-model analysis for association studies. Nat. Genet..

