# Supplementary figures and images for: PMEL governs autosomal dominant inheritance of white-tail independent of yellow body plumage in chickens (Gallus gallus domesticus)
Source: Poult Sci. 2025 Nov 16;105(1):106127. doi: 10.1016/j.psj.2025.106127 (PMC12671367; doi:10.1016/j.psj.2025.106127)

F<sub>0</sub>

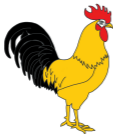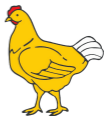

F<sub>1</sub>

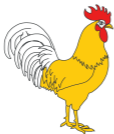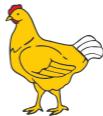

F<sub>2</sub>

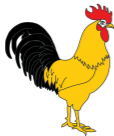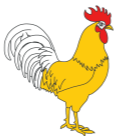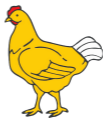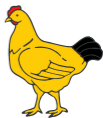

Supplement: Supplementary file 1 [file mmc1.pdf]

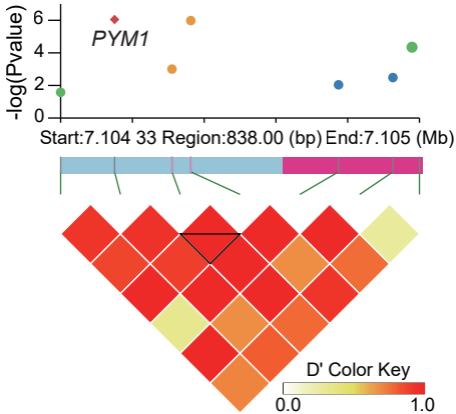

Supplement: Supplementary file 2 [file mmc2.pdf]
